# Identification of a PANoptosis-related Gene Signature for Predicting the Prognosis, Tumor Microenvironment and Therapy Response in Breast Cancer

**DOI:** 10.7150/jca.90113

**Published:** 2024-01-01

**Authors:** Tianshui Yu, Weilun Cheng, Jiarui Zhang, Ting Wang, Yansong Liu, Yunqiang Duan, Anbang Hu, Jianyuan Feng, Mingcui Li, Yanling Li, Hanyu Zhang, Zhiyuan Rong, Yuhang Shang, Suborna S. Shakila, Fei Ma, Baoliang Guo

**Affiliations:** Department of General Surgery, The Second Affiliated Hospital of Harbin Medical University, Harbin 150081, China.

**Keywords:** breast cancer, PANoptosis, prognostic model, tumor microenvironment, drug sensitivity, machine learning

## Abstract

Breast cancer (BC) is the most prevalent malignancy among women worldwide. Mounting evidence suggests that PANoptosis participates in cancer development and therapy. However, the role of PANoptosis in BC remains unclear. In this study, we identified ten PANoptosis-related genes using Cox regression analysis, random forest (RF) algorithm and least absolute shrinkage and selection operator (LASSO) algorithm. A PANoptosis-related score (PRS) was calculated based on the coefficient of LASSO. Notably, we divided the patients into high- and low-risk groups according to the PRS and revealed a negative correlation between PRS and overall survival. Next, a nomogram model was constructed and validated to improve the clinical application of PRS. Functional enrichment analyses and the Bayesian network demonstrated that differentially expressed genes between high- and low-risk groups were mainly enriched in immune-related pathways. Besides, we found significant differences in tumor mutation burden and tumor immune microenvironment between patients in these two groups using bulk-RNA and single-cell RNA sequencing data. Furthermore, charged multivesicular body protein 2B (CHMP2B) was identified as the hub gene by combining LASSO, weighted gene co-expression network analysis, RF and eXtreme Gradient Boosting. Importantly, using immunohistochemistry analysis based on our tissue microarray, we found that CHMP2B was highly expressed in tumor tissue, and CD4 and CD8 were more likely to be positive in the CHMP2B-negative group. Survival analyses revealed that CHMP2B adversely impacted the survival of BC patients. In conclusion, we not only constructed a highly accurate predictive model based on PRS, but also revealed the importance of PANoptosis-related gene signature in the modulation of the tumor microenvironment and drug sensitivity in BC.

## Introduction

Breast cancer (BC) is the most prevalent primary malignancy among women worldwide, accounting for 1 in 4 cancer cases and 1 in 6 cancer deaths of all cancers 1. Currently, BC treatment approaches mainly include surgery, chemotherapy, targeted therapy, endocrine therapy and radiotherapy 2. Considering the heterogeneity and complexity of BC, it is crucial to screen high-risk patients and implement more appropriate treatment strategies based on pathological characteristics 3. With the development of the DNA microarray and next-generation sequencing (NGS) over the past decades, individual gene signatures could provide alternative information to predict treatment sensitivity and prognosis of BC patients in addition to clinicopathological features 4-6.

Resistance to cell death is a hallmark of cancer 7. Cell death can be divided into accidental cell death (ACD) and regulated cell death (RCD), and the latter is generally referred to as programmed cell death (PCD) under physiological conditions 8. Apoptosis, pyroptosis and necroptosis are three critical PCD pathways characterized by particular molecular and genetic features 8. Many studies have already explicitly elucidated the importance of these pathways in the carcinogenesis and treatment of BC 9-11. Recently, based on the extensive cross-talk between these PCD pathways, a conceptualization of an integrated cell death modality called “PANoptosis” was formed 12. PANoptosis is an inflammatory PCD pathway activated by the simultaneous involvement of components from pyroptosis, apoptosis and necroptosis 13. It could not be characterized by any of these pathways alone, although it has the essential features of each of them 14. PANoptosis has been proven to participate in regulating the tumorigenesis of colorectal cancer and the immunotherapy response of gastric cancer 15, 16. Besides, He *et al.* found that PANoptosis was also related to the survival of BC patients 17. However, more comprehensive research is still needed to better unravel its role in modulating BC progression and therapeutic response, as well as the ability to predict the prognosis of BC patients in combination with other clinical information.

In this study, we constructed a nomogram based on PANoptosis-related genes and clinical features to predict the prognosis of BC patients for the first time, not only identifying the high-risk patients, but also helping to implement more appropriate treatment for certain patients.

## Materials and Methods

### Data source

The gene expression profile and related clinicopathological data of TCGA-BRCA were downloaded from UCSC Xena and utilized as the training cohort in this study 18. The METABRIC breast cancer data was downloaded from cBioPortal for external validation 19, 20. All of the patients enrolled had primary breast cancer and M0 tumor stage. The baseline characteristics are shown in Table S1 and compared using the chi-square test or Fisher's exact test. PANoptosis-related genes were created by combining the gene lists of pyroptosis, apoptosis and necroptosis, which were collected from the Molecular Signatures Database (MsigDB) and literature review 21. Single-cell RNA sequencing (scRNA-seq) data was extracted from the GSE161529 dataset in the Gene Expression Omnibus (GEO) database 22. One triple-negative breast cancer (TNBC), one human epidermal growth factor receptor 2-positive (HER2+) and one estrogen receptor-positive (ER+) cases were selected for the study. The workflow of this study is shown in Figure S1.

### Construction of PANoptosis-related risk score and nomogram

We used the Cox proportional hazard regression model to assess the association between the expression of each PANoptosis-related gene and the overall survival (OS) of patients in the TCGA-BRCA cohort. Afterward, the random forest (RF) algorithm was conducted to further screen featured genes using the randomForest R package 23. Genes with MeanDecreaseGini > 10 were selected for further analysis. The Least absolute shrinkage and selection operator (LASSO) algorithm was then applied to ensure the model's simplicity and minimize overfitting in the model training process 24. The risk score was constructed by using the regression coefficients derived from LASSO Cox regression analysis:



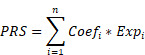



*PRS* means the PANoptosis-related score. *Coef_i_* and *Exp_i_* represent the coefficient and expression level of the corresponding gene, respectively. Based on the median risk score, all samples were separated into two categories: high-risk group (PRS > median value) and low-risk group (PRS < median value). Typical immunohistochemical (IHC) images of all model genes were downloaded from the Human Protein Atlas (HPA) database. The Kaplan-Meier (KM) analysis was used to compare the differences in OS between these two groups. The time-dependent receiver operating characteristic (ROC) curve was generated to evaluate the prognostic performance of the PRS. Combined with the clinicopathological features that were statistically significant in univariate (p-value < 0.10) and multivariate (p-value < 0.05) Cox regression analysis, we subsequently used the “rms” package to predict 1-, 3-, 5-, and 10-year OS by constructing a nomogram. The nomogram's discrimination performance was quantitatively assessed by the area under curve (AUC) of the ROC curve, and the calibration performance was evaluated by the calibration curve.

### Functional enrichment analysis

Differentially expressed genes (DEGs) between high- and low-risk groups were identified using the “DESeq2” package in R software 25. Functional enrichment analyses based on Kyoto Encyclopedia of Genes and Genomes (KEGG), Gene Ontology (GO) and Reactome databases were carried out between these two groups, and the results were evaluated by the R package “clusterProfiler” 26 and “ReactomePA” 27. The Bayesian network was applied using the “CBNplot” R package to understand how GO and Reactome pathways interact with one another 28.

### Somatic mutation analysis

Somatic mutation data retrieved from The Cancer Genome Atlas (TCGA) were analyzed using the R package “maftools” 29. The tumor mutational burden (TMB) of each patient between high- and low-risk groups was calculated and compared using the Wilcoxon rank-sum test.

### Immunogenomic landscape analysis

We used CIBERSORT to predict the proportions of 22 types of tumor-infiltrating immune cells (TIICs) in each sample. Single sample Gene Set Enrichment Analysis (ssGSEA) was applied to predict the abundance of 28 TIICs in individual tissue samples 30. Furthermore, we used the R package “estimate” to calculate each sample's ESTIMATE score. The Immunophenoscore (IPS) scores, which are calculated based on representative cell-type gene expression z-scores, were collected from the Cancer Immunome Atlas (TCIA) 31. The tumor immune exclusion score was calculated by the Tumor Immune Dysfunction and Exclusion (TIDE) 32, 33. Moreover, we compared the expression of genes from the human leukocyte antigen (HLA) family and several critical co-stimulators between high- and low-risk groups using the Wilcoxon rank-sum test.

### Identification of PANoptosis-associated single cells

Seurat (v4.3.0) was utilized for quality control and processing of scRNA-seq data 34. Cell types were annotated based on the CellMarker 2.0 database and relative literature 35, 36. Single-Cell Map of Diverse Immune Phenotypes in the Breast Tumor Microenvironment (Scissor) package was used to identify bulk phenotype-associated cell subpopulations 37. In this study, the input data of the Scissor pipeline included TCGA-BRCA bulk data, GSE161529 scRNA-seq data and PANoptosis-related risk group data obtained from LASSO analysis. Based on the signs of the estimated regression coefficients, the single cells were finally grouped into Scissor positive (Scissor+) and Scissor negative (Scissor-) cells, corresponding to the high-risk group and low-risk group in TCGA-BRCA bulk data, respectively.

### Therapeutic efficacy estimation

We utilized the R package “oncoPredict” to calculate the half-maximal inhibitory concentration (IC50) based on the GDSC2 dataset of the Genomics of Drug Sensitivity in Cancer (GDSC) database 38. Besides, we assessed the expression of target genes associated with drugs exhibiting different sensitivities between high- and low-risk groups using the DrugBank database 39.

### Screening for the hub gene

Weighted gene co-expression network analysis (WGCNA) was applied to identify hub genes among ten PANoptosis genes. We used the R package “WGCNA” to construct the co-expression network of genes from the TCGA-BRCA dataset 40. According to their expression patterns, genes were classified into four modules (brown, blue, turquoise and grey). Subsequently, the correlation between modules and PRS was calculated. The brown module, which showed the highest correlation with PRS, was selected for further analysis. Besides, we applied eXtreme Gradient Boosting (XGBoost) to further calculate the importance of each gene in this ten-gene signature using the R package “XGBoost” 41. Finally, the hub gene was characterized by combining the results from WGCNA, LASSO, RF and XGBoost.

### Validation of the hub gene by IHC

The tissue microarray (TMA) was made by the production company (SHANGHAI OUTDO BIOTECH CO., LTD.) using wax blocks of 105 breast cancer and 41 normal breast tissue. The tissues were collected from patients with primary breast cancer and without metastases at diagnosis in the Second Affiliated Hospital of Harbin Medical University (Table S1). After de-paraffinization and rehydration, tissue sections were incubated in antigen retrieval buffer and heated in a steamer above 97 °C for 20 min. IHC staining was conducted utilizing Ventana Discovery XT Automated Slide Stainer (Ventana Medical Systems, Inc., Tucson, AZ, USA). The automated Discovery XT system was employed to carry out deparaffinization, antigen retrieval, blocking, DAB detection, counterstaining, post-counterstaining and slide cleaning. Charged multivesicular body protein 2B (CHMP2B) antibody (Abclonal, A19244), CD4 antibody (MXB biotechnologies, RMA-0620) and CD8 antibody (MXB biotechnologies, MAB-1031) were respectively applied to tissue sections overnight in a humidity chamber at a dilution of 1:200 at 4 °C. After washing in TBS, the antigen-antibody binding was detected using the Envision+ system and DAB+ chromogen (DAKO). Tissue sections were briefly immersed in hematoxylin for counterstaining, washed with water and covered with coverslips. Subsequently, slides were independently evaluated by two pathologists under a multi-headed microscope in a blinded manner. The staining intensity level was scored from 0 to 3 (no staining, light brown, brown, and tan). The staining extent was rated from 0 to 4 based on the percentage of positive cells (0-5%, 5-25%, 26-50%, 51-75%, and 76-100%). Finally, the IHC score was defined as the product of staining intensity and extent scores, ranging from 0 to 12. Scores 0-4 were grouped as negative and 5-12 as positive. Clinical characteristics were compared using Chi-square tests. Continuous variables were converted to categorical using X-tile software (Version 3.6.1) 42. We then used the KM curve to estimate survival outcomes, and the Cox proportional hazards model was performed to fit clinical characteristics for OS. This research was approved by the Ethics Committee of Harbin Medical University (reference number KY2021-058).

### Statistical analysis

All statistical analyses were implemented using R v4.2.2 and SPSS 26.0. A p-value < 0.05 was considered significant unless stated otherwise.

## Results

### Identification of a ten-gene PANoptosis-related signature in breast cancer

**Figure 1:**
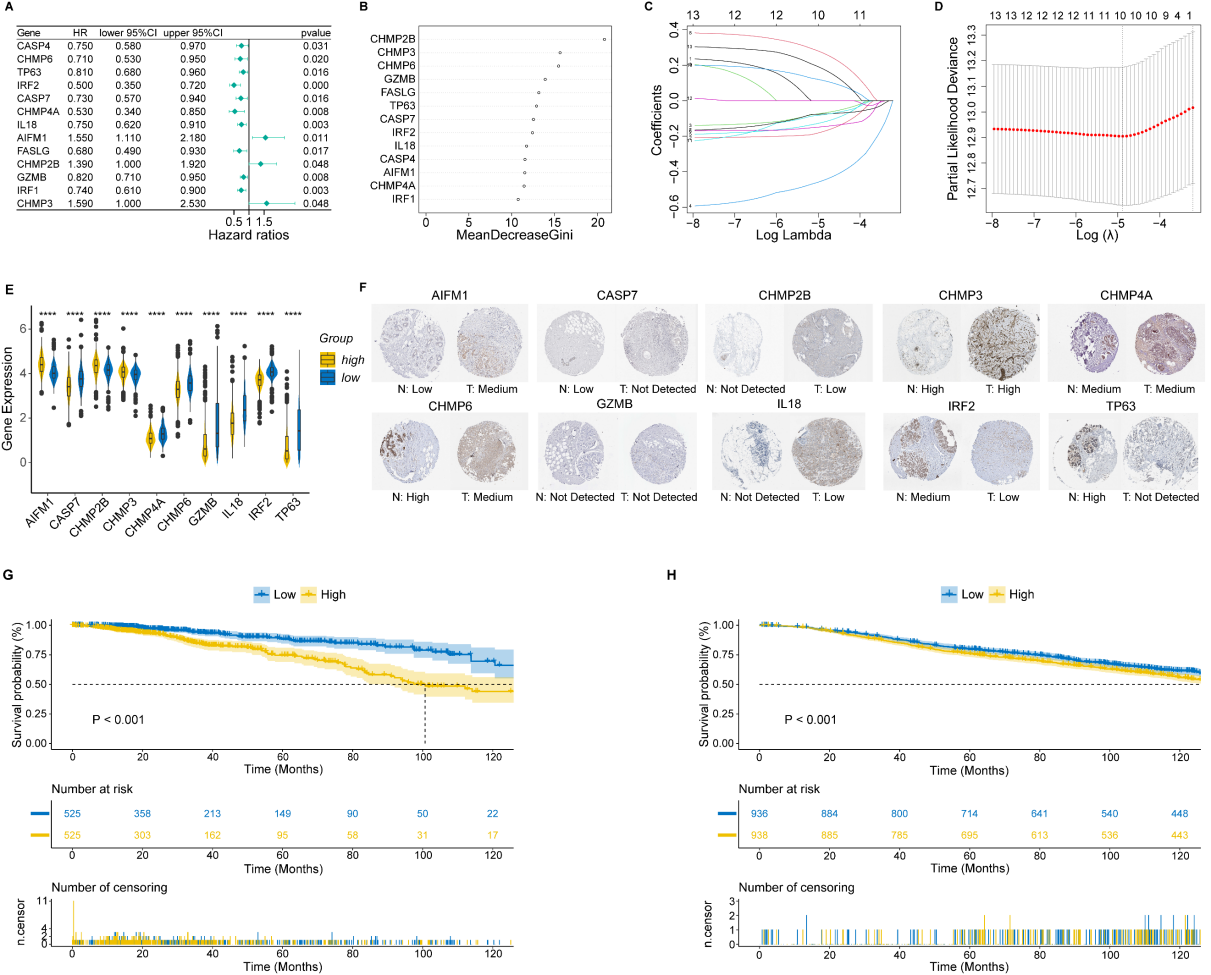
Identification of a ten-gene PANoptosis-related signature. **(A)** Forest plot of statistically significant genes in the univariate Cox regression analysis; **(B)** The MeanDecreaseGini index for the thirteen PANoptosis-related genes in the random forest (RF) analysis. **(C)** Coefficient profiles of PANoptosis-related genes in the least absolute shrinkage and selection operator (LASSO); **(D)** identification of the best parameter (lambda) in LASSO; **(E)** Violin plot of the expression of ten PANoptosis-related genes in high and low PRS groups; **(F)** typical immunohistochemical (IHC) images of ten PANoptosis-related genes in normal and breast tumor tissues from the HPA database;** (G)** Kaplan-Meier (KM) analysis based on PANoptosis-related score (PRS) in the training cohort; **(H)** KM analysis based on PRS in the validation cohort. HR: hazard ratio; CI: confidence interval; high: high-risk group; low: low-risk group; N: normal; T: tumor; ****: p < 0.0001.

Sixty-five PANoptosis-related genes (Table S2) were input into univariate Cox regression analysis in the TCGA-BRCA training set, and thirteen genes significantly affected OS were obtained (Figure 1A). Subsequently, we applied the RF analysis and found that the MeanDecreaseGini for each of these genes was higher than 10 (Figure 1B). Then, the LASSO regression analysis was performed on these genes, and ten PANoptosis-related genes with non-zero coefficients were selected for further model construction (Figure 1C, D). Finally, based on the coefficients derived from the LASSO analysis, the formula for the risk score was as follows: PRS = (-0.174034262515715)*CHMP6 + (-0.0701780204898664)*TP63 + (-0.449396144853978)*IRF2 + (-0.121272009866694)*CASP7 + (-0.146272576423145)*CHMP4A + (-0.0749366454708963)*IL18 + 0.268668804297125*AIFM1 + 0.114110313114337*CHMP2B + (-0.0789991578059556)*GZMB + 0.179084139171232*CHMP3. To better unearth the potential mechanism of PANoptosis-related genes, we divided patients into high-risk and low-risk groups based on their PRS. All ten PANoptosis-related genes used for model construction were differentially expressed between the high- and low-risk groups (Figure 1E). Besides, we obtained typical IHC images of these genes from the HPA database (Figure 1F). Notably, the KM analysis further proved the prognostic value of PRS (Figure 1G). For external validation, the result of METABRIC data was consistent with the TCGA cohort (Figure 1H).

### Construction and validation of a clinical nomogram

**Figure 2:**
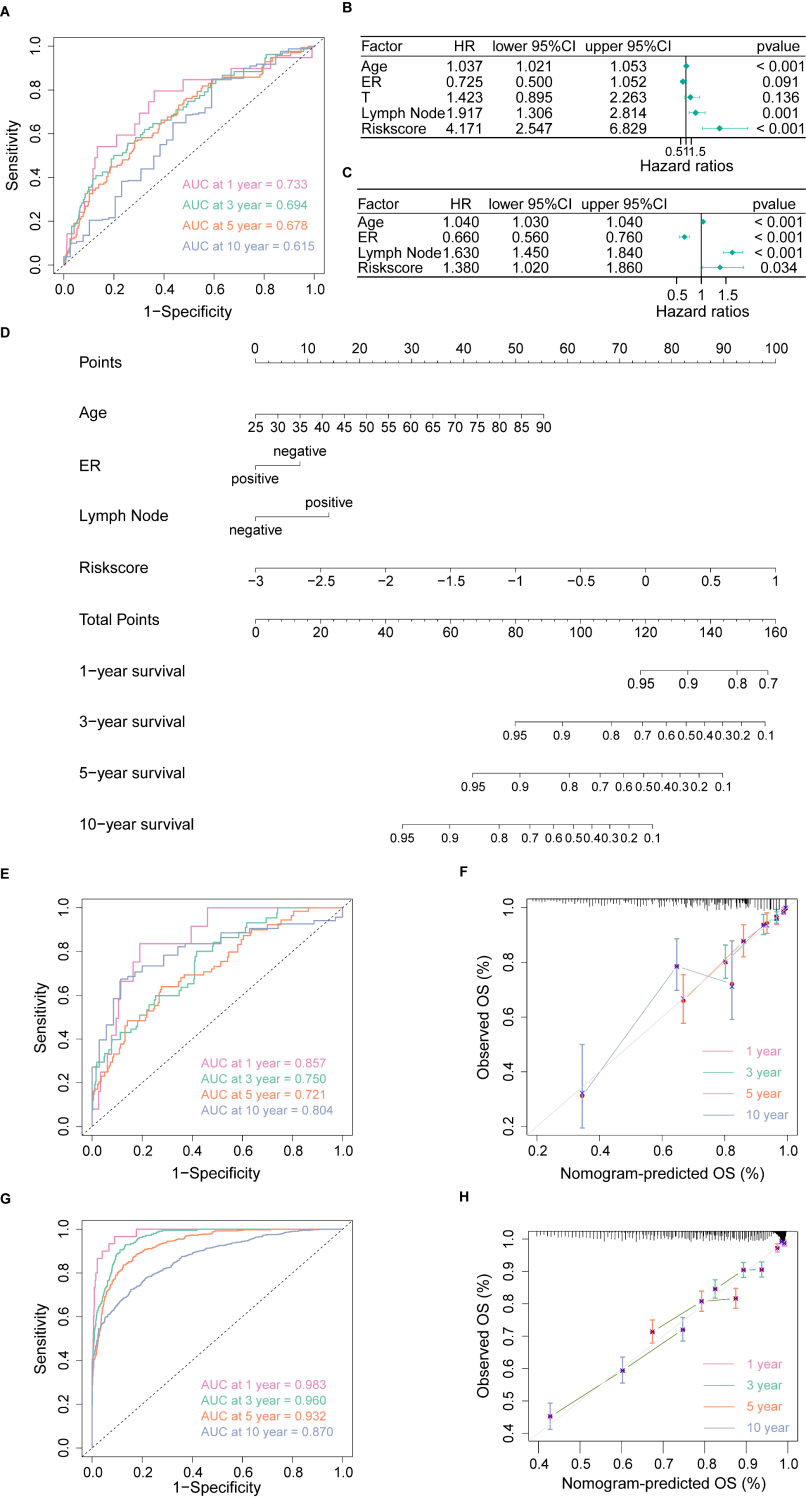
Construction and validation of a clinical nomogram. **(A)** Time-dependent receiver operating characteristic (ROC) curves of the PANoptosis-related score (PRS) in predicting the 1-, 3-, 5- and 10-year overall survival (OS); **(B)** Univariate Cox regression analysis of PRS and clinical characteristics; **(C)** Multivariate Cox regression analysis of PRS and clinical characteristics; **(D)** Nomogram based on the PRS, age, ER status and lymph node status; **(E)** Time-dependent ROC curves of the nomogram in predicting the 1-, 3-, 5- and 10-year OS in the training cohort; **(F)** Calibration curves of the nomogram in predicting the 1-, 3-, 5- and 10-year OS in the training cohort; **(G)** Time-dependent ROC curves of the nomogram in predicting the 1-, 3-, 5- and 10-year OS in the validation cohort; **(H)** Calibration curves of the nomogram in predicting the 1-, 3-, 5- and 10-year OS in the validation cohort. AUC, area under curve; HR: hazard ratio; CI: confidence interval; ER, estrogen receptor; OS, overall survival.

To develop a nomogram based on PRS and clinical features, we first applied time-dependent ROC to evaluate the prediction efficiency of PRS. As shown in Figure 2A, the AUC at 1-year, 3-year, 5-year and 10-year OS was 0.733, 0.694, 0.678 and 0.615, respectively. Next, we evaluated the prognostic effect of PRS, age, estrogen receptor (ER) status, T stage and lymph node status in univariate Cox regression analysis, and factors with p-value < 0.1 were enrolled in multivariate Cox regression analysis (Figure 2B). According to the results of multivariate Cox regression analysis, we selected age, ER status, lymph node status and PRS to develop the final model (Figure 2C). Given the complexity of the risk score formula, we constructed a nomogram to predict 1-, 3-, 5- and 10-year OS of BC patients (Figure 2D). In our model, higher OS rates were associated with a lower risk score, younger age, positive ER status and negative lymph node metastasis. The AUC of the nomogram was 0.857, 0.750, 0.721, and 0.804 for 1-, 3-, 5- and 10-year survival, respectively (Figure 2E). The calibration curve revealed that the predicted curve of the nomogram was nearly identical to the actual curve of BC patients, suggesting the close relationship between predicted survival rates and actual rates at 1, 3, 5 and 10 years (Figure 2F). In addition, the ROC and calibration curves of the external validation cohort further confirmed the significant predictive value of our model (Figure 2G, H). Taken together, these results demonstrated the outstanding performance of our nomogram compared with previously established prognostic models 43-45.

### Identification of PANoptosis-related signaling pathways

**Figure 3:**
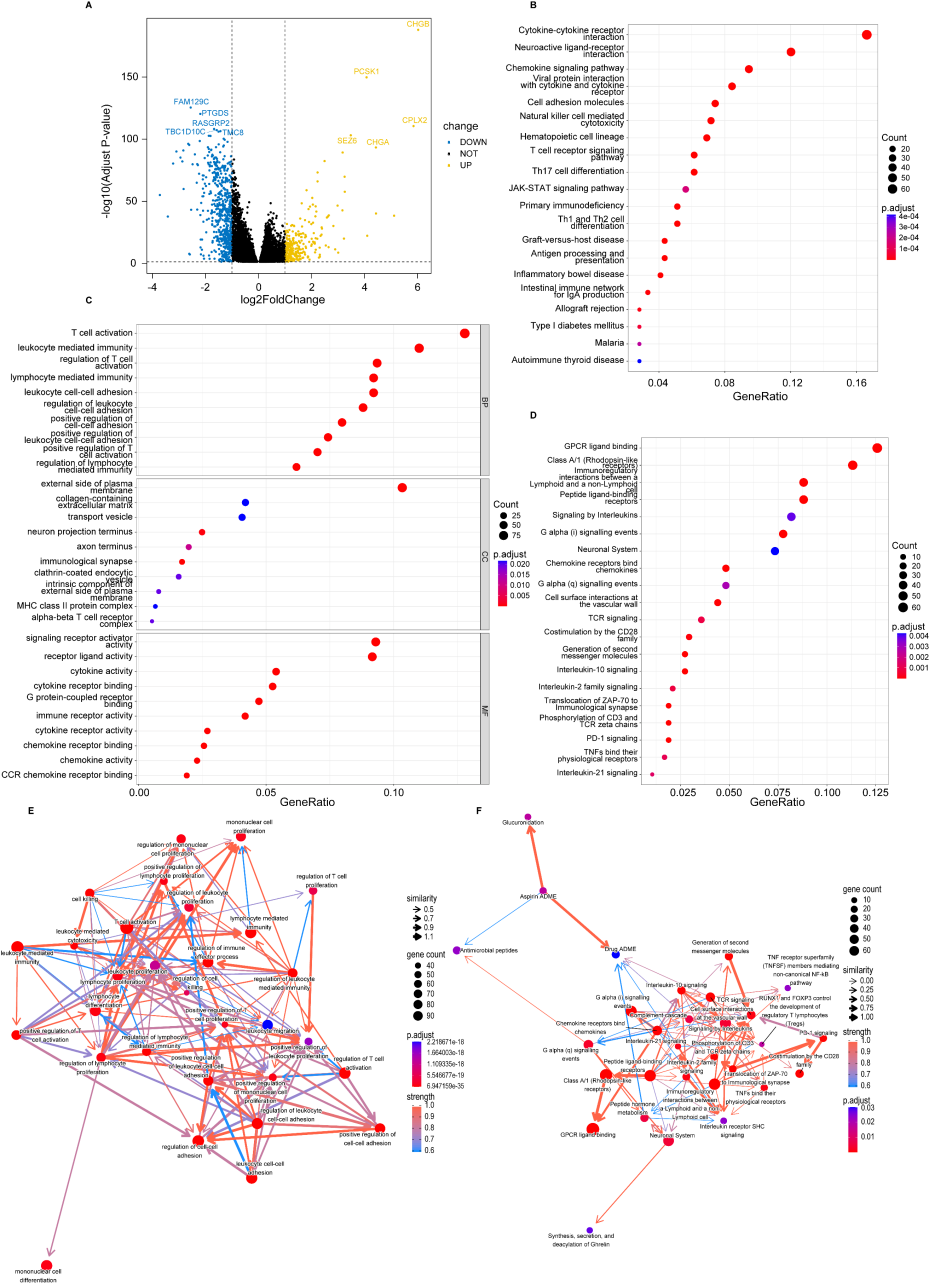
Identification of PANoptosis-related signaling pathways. **(A)** Volcano plot of DEGs based on PRS; **(B)** Dot plot of Kyoto Encyclopedia of Genes and Genomes (KEGG) enrichment analysis; **(C)** Dot plot of Gene Ontology (GO) enrichment analysis;** (D)** Dot plot of Reactome enrichment analysis;** (E)** Bayesian network (BN) based on GO analysis; **(F)** Bayesian network (BN) based on Reactome analysis.

We found 843 DEGs, including 275 up-regulated genes and 568 down-regulated genes with |log_2_FC| > 1 and adjusted p-value < 0.05 between high- and low-risk groups (Figure 3A). In functional enrichment analysis, DEGs were mainly enriched in pathways correlated to immunity, including cytokine-cytokine receptor interaction, natural killer cell mediated cytotoxicity, T cell receptor signaling pathway, and Th1, Th2 and Th17 cell differentiation in KEGG analysis (Figure 3B, Table S3).

GO analysis also revealed that DEGs were enriched in gene sets associated with T cell activation, lymphocyte mediated immunity, leukocyte cell-cell adhesion and immune receptor activity (Figure 3C, Table S4). Besides, enrichment based on the Reactome database demonstrated that DEGs were related to G protein-coupled receptor (GPCR) ligand binding, signaling by interleukins, T cell antigen receptor (TCR) signaling and PD-1 signaling (Figure 3D, Table S5). Furthermore, the BN showed that most pathways were intensely correlated (Figure 3E, F).

### Tumor mutation analysis

**Figure 4:**
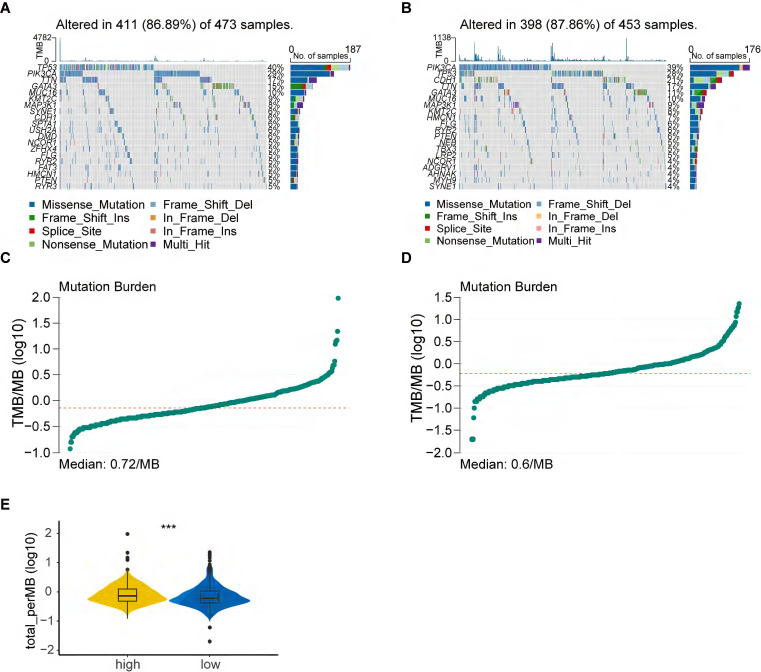
Tumor mutation analysis. **(A)** Waterfall plot of somatic mutation features established with high PANoptosis-related score (PRS); **(B)** Waterfall plot of somatic mutation features established with low PRS; **(C)** tumor mutation burden (TMB) in the high-risk group; **(D)** TMB in the low-risk group; **(E)** Violin plot of TMB in the high- and low-risk groups. high: high-risk group; low: low-risk group; ***, p < 0.001.

Considering that genetic mutations are crucial factors in tumorigenesis, we estimated the situation of somatic mutation between two groups. The somatic mutation rate of the high-risk group was 86.89% (411 of 473 samples), primarily the missense mutation, and TP53 showed the highest frequency of mutations (40%) (Figure 4A). The mutation rate in the low-risk group was 87.86% (398 of 453 samples), mainly the missense mutation, and PIK3CA demonstrated the highest frequency of mutations (39%) (Figure 4B). In addition, TMB quantification analysis revealed a higher TMB in the high-risk group (Figure 4C-E).

### Comprehensive analysis of tumor microenvironment

**Figure 5:**
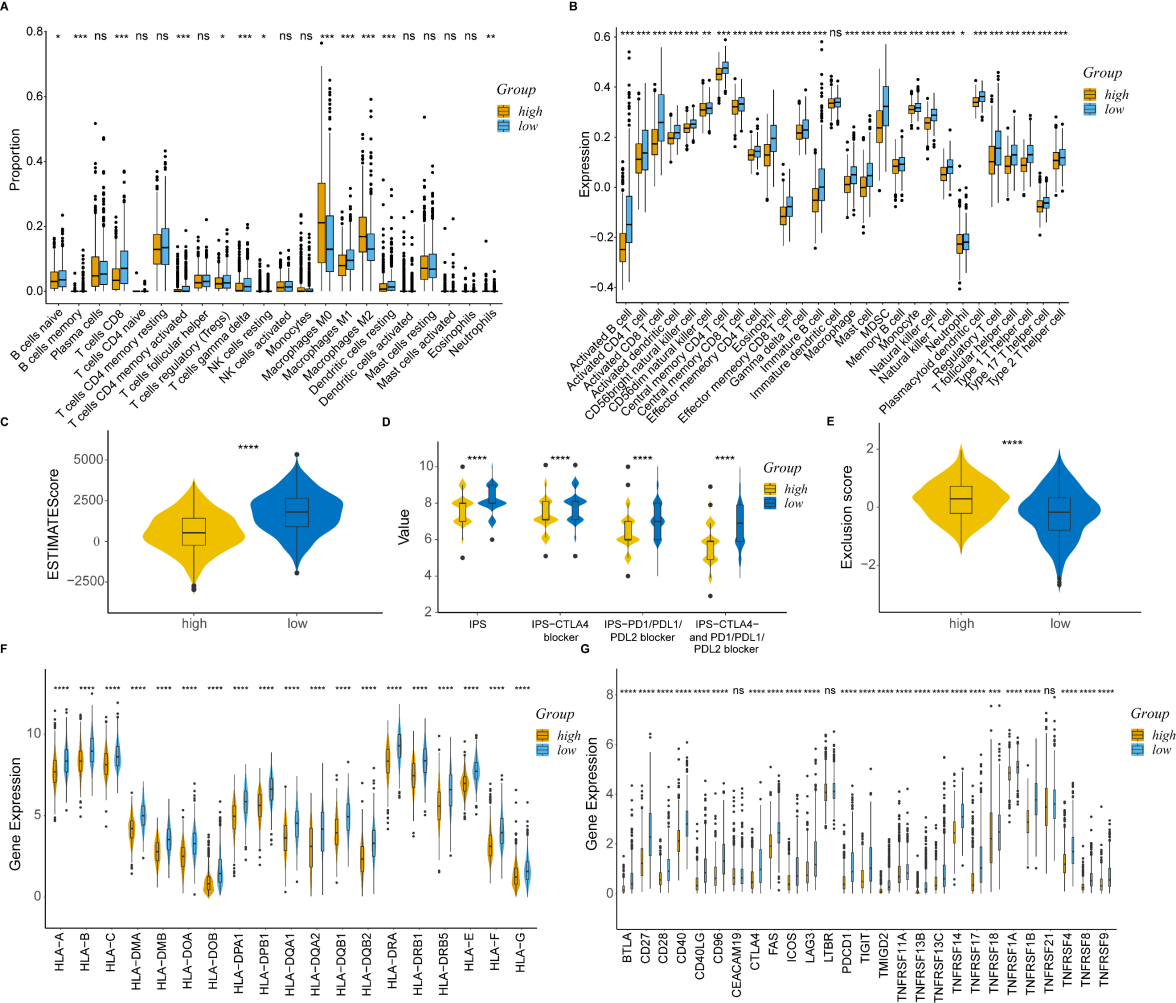
Comprehensive analysis of tumor immune microenvironment. **(A)** Boxplot of immune cell proportion in the high- and low-risk groups calculated by CIBERSORT algorithm; **(B)** Boxplot of immune cell expression in the high- and low-risk groups calculated by ssGSEA algorithm; **(C)** Violin plot of ESTIMATE score in the high- and low-risk groups; **(D)** Violin plot of IPS in the high- and low-risk groups; **(E)** Violin plot of TIDE exclusion score in the high- and low-risk groups; **(F)** Violin plot of the expression levels of HLA molecules; **(G)** Violin plot of the expression levels of co-stimulators. high: high-risk group; low: low-risk group; IPS, Immunophenoscore; ns, non-significant; *, p < 0.05; **, p < 0.01; ***, p < 0.001; ****, p < 0.0001.

To delineate the immune status of the tumor microenvironment (TME), we performed the CIBERSORT algorithm to calculate the proportion of immune cells in each group (Figure 5A).

As expected, the high-risk group was associated with saliently fewer CD8+ T cells, M1 macrophages and more M2 macrophages than the low-risk group. ssGSEA also demonstrated a lower expression of most TIICs, including activated B cell, CD4+ T cell, CD8+ T cell and dendritic cell in the high-risk group (Figure 5B). Furthermore, the immune score calculated by the ESTIMATE algorithm was statistically lower in the high-risk group (Figure 5C). The results obtained from TCIA illustrated that the relative probabilities of responding to immunotherapy in the high-risk group were lower than those in the low-risk group, regardless of CTLA4 and PD1 status (Figure 5D). The high-risk group also showed a higher tumor immune exclusion score, indicating an immune suppressive microenvironment (Figure 5E). Moreover, most of the HLA genes (Figure 5F) and immune checkpoints (Figure 5G) were considerably higher expressed in the low-risk group.

### PANoptosis-associated single cell analysis

**Figure 6:**
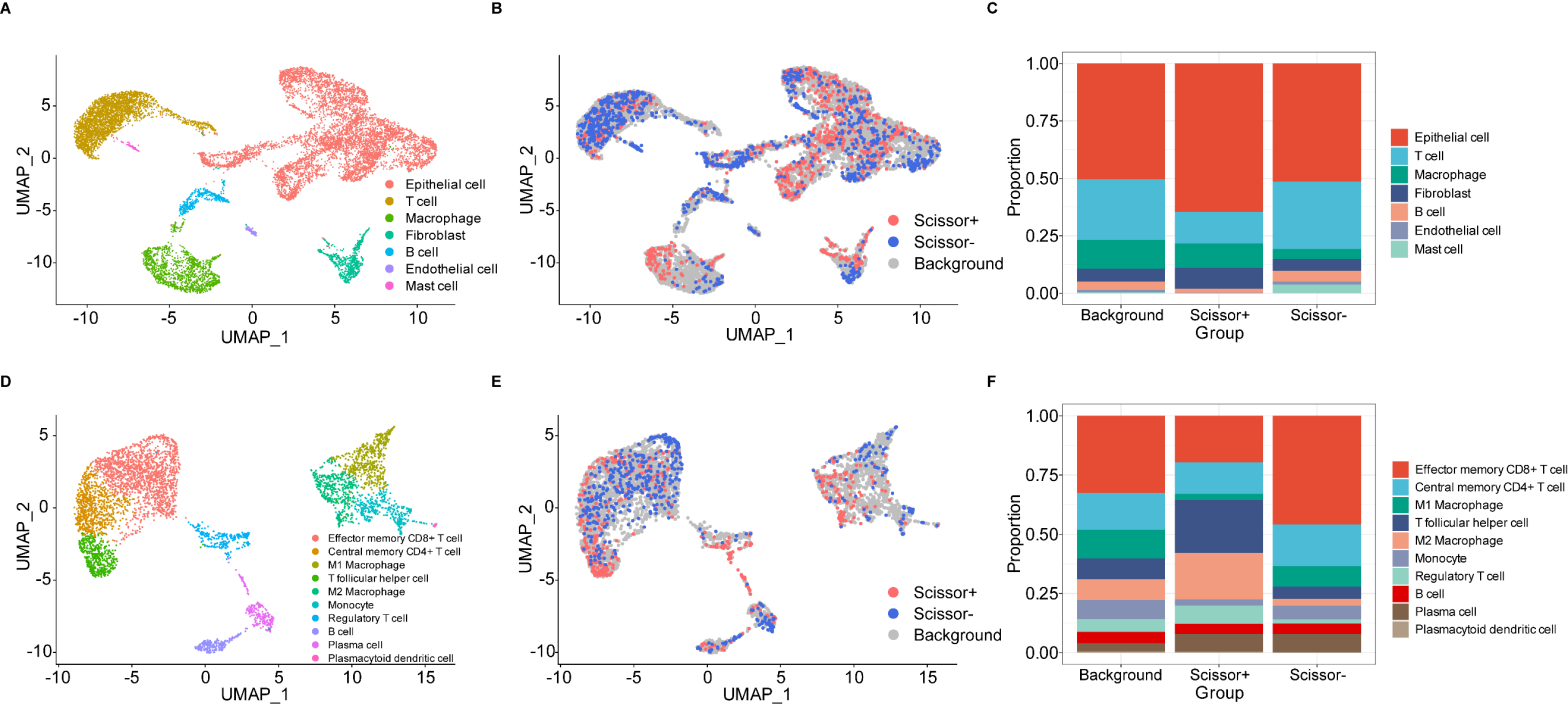
PANoptosis-associated TME analysis at single-cell resolution. (**A**) UMAP visualization of cell-type-specific annotation. (**B**) UMAP visualization of Scissor+ and Scissor- cells. (**C**) Proportional fractions of identified cell types across Scissor+/- condition. (**D**) UMAP visualization of cell-type-specific annotation among extracted immune cells. (**E**) UMAP visualization of Scissor+ and Scissor- cells among extracted immune cells. (**F**) Proportional fractions of identified cell types across Scissor+/- condition among extracted immune cells.

We utilized the standard Seurat pipeline to explore the scRNA-seq data and got seven cell types after dimension reduction, cell clustering and annotation (Figure S2A, B, Figure 6A). Subsequently, the Scissor algorithm was applied to identify the PANoptosis-associated cell subpopulations. In this analysis, 884 Scissor+ cells (corresponding to the high-risk group) and 1021 Scissor- cells (corresponding to the low-risk group) were classified by the PRS of bulk samples (Figure 6B). Notably, we found that proportional fractions of T cell, B cell, mast cell and endothelial cell in the Scissor- group were higher than Scissor+ group, while epithelial cell, macrophage and fibroblast were lower (Figure 6C). Then, we extracted T cells, B cells and macrophages to further evaluate the relationship between PANoptosis and tumor immune microenvironment. In the subgroup analysis, immune cells were classified into ten types (Figure S2C, D, Figure 6D). Similarly, we got 458 Scissor+ cells and 520 Scissor- cells using the Scissor algorithm (Figure 6E). Concordantly, the proportion of cell types demonstrated that proportional fractions of effector memory CD8+ T cell, central memory CD4+ T cell, M1 macrophage, monocyte, B cell and plasma cell in the Scissor- group were higher than Scissor+ group, while T follicular helper cell, M2 macrophage, regulatory T cell and plasmacytoid dendritic cell were lower (Figure 6F). These results were consistent with our immune infiltration analysis using bulk data, further validating the positive correlation between higher PRS (high-risk group) and tumor-promoting immune microenvironment.

### Drug sensitivity analysis

**Figure 7:**
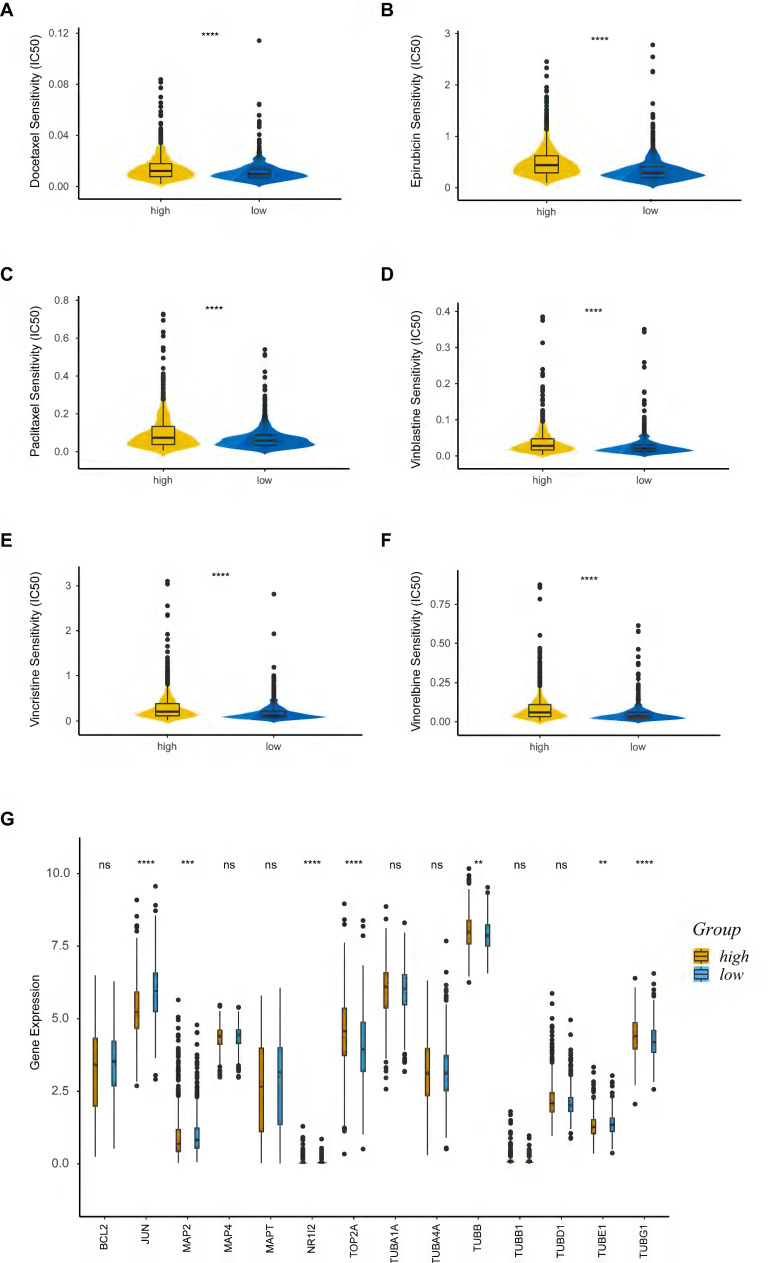
Drug sensitivity analysis. **(A-F)** Sensitivity analysis for Docetaxel **(A)**, Epirubicin **(B)**, Paclitaxel **(C)**, Vinblastine **(D)**, Vincristine **(E)** and Vinorelbine **(F)** in patients at low and high risk; **(G)** Boxplot of target genes of differentially sensitive drugs. IC50, half-maximal inhibitory concentration; ns, non-significant; high: high-risk group; low: low-risk group; **, p < 0.01; ***, p < 0.001; ****, p < 0.0001.

We explored the association between PRS and anticancer drug sensitivity in the GDSC database. Strikingly, we found some commonly used anticancer chemotherapeutic drug agents, including docetaxel (Figure 7A), epirubicin (Figure 7B), paclitaxel (Figure 7C), vinblastine (Figure 7D), vincristine (Figure 7E) and vinorelbine (Figure 7F), had a lower IC50 in the low-risk group, indicating their better responses in treating the low-PRS patients. We further assessed the expressions of target genes associated with the drugs mentioned above. Target genes from the DrugBank database, included JUN, MAP2, NR1I2, TOP2A, TUBB, TUBE1 and TUBG1, were differentially expressed between the high-risk and low-risk groups (Figure 7G). These results suggested that PRS could identify more suitable patients for appropriate anticancer drug therapy.

### Hub gene screening and experimental verification

To identify the most crucial hub gene among ten model genes, we first initiated the WGCNA algorithm. A weighted gene co-expression network was established utilizing the TCGA-BRCA dataset (Figure S3A). The scale-free network was constructed by setting the soft threshold to 6 (Figure S3B). Moreover, we created an adjacency matrix and converted it into a Topological Overlap Matrix (TOM). Four gene modules were identified according to the TOM, namely blue (3465), brown (2684), grey (2599), and turquoise (10775) modules. We found that the brown module exhibited the strongest correlation with PRS (Figure S3C). Next, we extracted the overlapping genes between the brown module and the ten model genes, resulting in the discovery of four genes - CHMP2B, CHMP3, CHMP4A, and CHMP6. Finally, after incorporating the findings from the RF (Figure 1B) and XGBoost (Figure S3D) analysis, we selected CHMP2B as the hub gene for further experimental verification.

**Figure 8:**
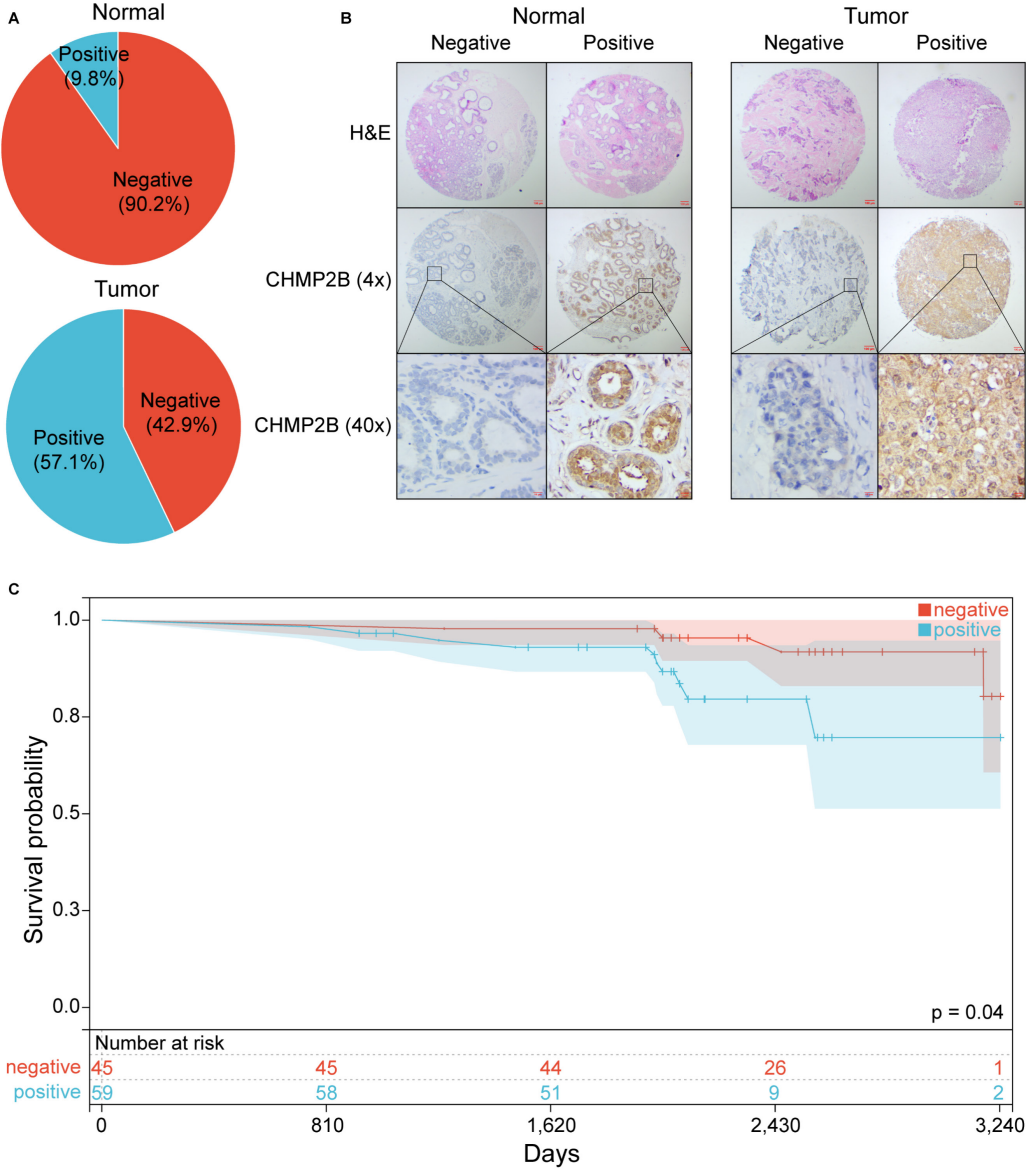
Experimental validation using IHC analysis. (**A**) Pie chart of CHMP2B expression in normal and breast tumor tissue. (**B**) Representative images of CHMP2B expression in normal (left panel) and tumor (right panel) tissue. (**C**) KM survival analysis between patients in CHMP2B-negative and CHMP2B-positive groups. IHC, immunohistochemistry; KM, Kaplan-Meier.

We then performed IHC analyses based on a TMA made by 105 BC and 41 normal breast tissue. In this cohort, 57.1% of BC patients were CHMP2B positive, while only 9.8% of healthy individuals highly expressed CHMP2B (Figure 8A, Table 1).

BC patients were then assigned to the CHMP2B-positive or CHMP2B-negative group according to their IHC scores. Unsurprisingly, we found that CD4 and CD8 were more likely to be positive in the CHMP2B-negative group, which was consistent with our former bioinformatical findings (Table 2).

Typical photos of IHC in our cohort are shown in Figure 8B and Figure S4. Notably, the KM plot illustrated that patients in the CHMP2B-negative group shared better prognoses than those in the CHMP2B-positive group (Figure 8C). The univariate and multivariate Cox analysis further confirmed that CHMP2B was a detrimental prognostic factor in breast cancer (Table 3).

## Discussion

Several types of PCD pathways, including apoptosis, necroptosis and pyroptosis, have been found to play an essential regulatory role in cancer development 46. Apoptosis is generally divided into an intrinsic pathway and an extrinsic pathway, both mediated by the activation of initiator and executioner caspases 47. Activation of necroptosis is mediated by the RIP kinases RIPK1 and RIPK3, together with the pore-forming pseudokinase MLKL, downstream of necrosome formation 48, 49. Pyroptosis is activated through the inflammatory caspases (caspase-1 and caspase-4/5) and executed by the gasdermin family members (GSDMD and GSDME) 50. PANoptosis is an integrated system in which any of the three PCD pathways can compensate for one another and work together at different times based on the context of the stimulus provided 14. It is regulated by the PANoptosome complex, a molecular scaffold for engaging pivotal pyroptotic, apoptotic, and necroptotic machinery 12. In oncology research, PANoptosis has been found to regulate the tumorigenesis of colorectal cancer and the immunotherapy response of gastric cancer 15, 16. Additionally, PANoptosis-related genes could also predict the survival of BC patients and modulate tumor immune microenvironment 17. Moreover, Maitra *et al.* reviewed the molecules in the PANoptosis pathway and proposed the hypothesis that PD-1/PD-L1-targeted inhibitor might play its role through PANoptosis pathway, but the specific mechanism of PANoptosis in breast cancer still needs further exploration 51.

Despite the similar research topic 17, this study screened PANoptosis-related genes using the combination of Cox regression, random forest and LASSO algorithm and built a highly accurate nomogram based on PANoptosis gene signature and clinicopathological information for the first time. Among genes in our model, a previous study has shown that tumor protein p63 (TP63) could act both as a tumor suppressor and an oncogene in breast cancer, depending on the cellular context 52. Moreover, Interleukin 18 (IL-18), a pro-inflammatory cytokine, is up-regulated on tumor-infiltrating lymphocytes, suggesting that IL-18 therapy could enhance anti-tumor immunity 53.

Recently, several models have been developed to predict the prognosis of BC patients. Tian *et al.* have built a prognostic model using basement membrane-related genes 43. Cui *et al.* have also established a nomogram based on nicotinamide metabolism-related signature 44, while Li's model was constructed utilizing m6A-related genes 45. However, none of these models perform better than our PANoptosis-based nomogram (assessed using AUC), further proving the vital value of our research.

We not only constructed a PRS-based prognostic model, but also conducted a series of comprehensive analyses between patients in the high- and low-risk groups. We found that DEGs were enriched in several pathways associated with immunity in the functional enrichment analysis. Moreover, BN demonstrated an intense correlation among these pathways. These results suggested that different survival outcomes between patients in high- and low-risk groups might be partially due to different tumor immune microenvironments. To further confirm this conjecture, we conducted TMB and immune infiltration analyses.

TMB is a quintessential predictive marker for cancer immunotherapy 54. In this study, we found that the high-risk group had higher TMB scores. Namely, patients with higher PRS might benefit more from immunotherapy than those with low PRS. We also uncovered the association between PRS and immune cell infiltration using various approaches. According to our research, the PRS was positively related to M0 and M2 macrophages and inversely associated with most other immune cells. Importantly, we utilized the “Scissor” algorithm to better illustrate the differences in immune infiltration between high and low PRS groups at the single-cell level. Concordant with former results, we found that effector memory CD8+ T cell, central memory CD4+ T cell, M1 macrophage, monocyte, B cell and plasma cell were enriched in the Scissor- (low-risk) group, while T follicular helper cell, M2 macrophage, regulatory T cell and plasmacytoid dendritic cell were more abundant in the Scissor+ (high-risk) group. Furthermore, patients in the low-risk group were more sensitive to commonly used chemotherapeutic drugs including docetaxel, epirubicin, paclitaxel, vinblastine, vincristine and vinorelbine. All of these results are in alignment with the better survival outcomes of patients in the low-risk group.

Notably, we identified CHMP2B as the hub gene among ten PANoptosis-related genes combining LASSO, WGCNA, RF and XGBoost methods. The role of CHMP2B in cancer is still controversial. On the one hand, it has a detrimental effect on the survival of patients with pancreatic adenocarcinoma 55, and overexpression of cPLA2G4A and CHMP2B simultaneously is correlated to the higher grade of myxofibrosarcoma and myxoid liposarcoma 56. On the other hand, CHMP2B is decreased in patients with endometrial carcinoma and the urinary exosomes of patients with colorectal cancer compared to healthy individuals 57, 58. Recently, Guo *et al.* revealed that cytoplasmic Yes1-associated transcriptional regulator (YAP1) could inhibit the proliferation of breast tumors by promoting autophagy, potentially through the combination of CHMP2B and VPS4B 59. However, the experiments conducted and the model constructed in this research were mainly based on YAP1, while the role of CHMP2B itself remained to be further explored. In our study, we applied IHC analysis based on tissue microarray to further validate the role of CHMP2B in breast cancer. The results indicated that patients with highly expressed CHMP2B had significantly lower expressions of CD4 and CD8 and worse prognoses, which were consistent with bioinformatic analyses.

Despite the merits of our findings, several limitations remain to be noted. First, our model was constructed and validated retrospectively. Hence, prospective research in the real world is highly required to underpin the clinical utility of our model. Additionally, although IHC analysis based on tissue array was performed, rational and precise mechanical experiments are considered necessary to unravel the underlying mechanisms of these PANoptosis-related genes.

In conclusion, our study constructed and validated a PANoptosis-based prognostic model, which provided significant value in predicting the survival outcomes of BC patients. Besides, we processed a series of comprehensive analyses between patients with high and low PRS, further confirming the importance of PANoptosis-related gene signature in the modulation of TME and drug sensitivity in BC, providing pivotal insights for subsequent mechanical research and helping clinicians make more personalized treatment decisions.

## Supplementary Material

Supplementary figures and tables.

## Figures and Tables

**Table 1:** Comparison of CHMP2B expression between BC patients and health controls.

	BC (n = 105)		HC (n = 41)		p-value
	N	%	N	%	
CHMP2B					**< 0.001**
Negative	45	42.9	37	90.2	
Positive	60	57.1	4	9.8	

BC, breast cancer; HC, health control; N, number; %, percentage.

**Table 2:** Relationship between CHMP2B expression and clinicopathological characteristics of BC patients.

Variables	CHMP2B				
	Positive (n = 60)		Negative (n = 45)		p-value
	*^a^*N	%	N	%	
Age, years					0.299
33-51	34	56.7	30	66.7	
52-83	26	43.3	15	33.3	
*^b^*N status					0.525
Negative	35	58.3	29	64.4	
Positive	25	41.7	16	35.6	
Subtype					0.212
Luminal	30	50.0	30	66.7	
HER2+	20	33.3	11	24.4	
TNBC	10	16.7	4	8.9	
Ki-67					0.139
< 14%	9	15.0	12	26.7	
≥ 14%	51	85.0	33	73.3	
CD4					**0.024**
Negative	47	78.3	26	57.8	
Positive	13	21.7	19	42.2	
CD8					**0.017**
Negative	57	95.0	36	80.0	
Positive	3	5.0	9	20.0	

BC, breast cancer; *^a^*N, number; %, percentage; ***^b^***N, lymph node; HER2+, human epidermal growth factor receptor 2-positive; TNBC, triple-negative breast cancer.

**Table 3:** Univariate and multivariate Cox analysis of prognostic factors among BC patients.

	Univariate Analysis			Multivariate Analysis		
	HR	95% CI	p-value	HR	95% CI	p-value
Age, years						
33-51 vs. 52-83	1.281	0.437-3.753	0.651			
N status						
Positive vs. Negative	3.017	1.070-8.510	0.037	2.833	1.001-8.021	0.050
Subtype			0.037			0.220
HER2+ vs. Luminal	4.763	1.414-16.046	0.012	-	-	0.200
TNBC vs. Luminal	4.017	0.876-18.427	0.074	-	-	0.516
ki-67						
< 14% vs. > 14%	3.321	0.691-15.962	0.134			
CHMP2B						
Positive vs. Negative	3.832	1.166-12.590	0.027	3.546	1.095-11.480	0.035

HR, hazard ratio; CI, confidence interval; N, lymph node; TNBC, triple-negative breast cancer; HER2+, human epidermal growth factor receptor 2-positive.

## Abbreviations

BC: breast cancer
RF: random forest
LASSO: Least absolute shrinkage and selection operator
PRS: PANoptosis-related score
CHMP2B: charged multivesicular body protein 2B
scRNA-seq: single-cell RNA sequencing
GEO: Gene Expression Omnibus
TNBC: triple-negative breast cancer
HER2+: human epidermal growth factor receptor 2-positive
ER+: estrogen receptor-positive
OS: overall survival
BN: Bayesian network
DEGs: differentially expressed genes
TMB: tumor mutation burden
IPS: Immunophenoscore
WGCNA: weighted gene co-expression network analysis
IHC: immunohistochemistry
TMA: tissue microarray
CHMP6: charged multivesicular body protein 6
TP63: tumor protein p63
IRF2: Interferon regulatory factor 2
CASP7: caspase 7
CHMP4A: charged multivesicular body protein 4A
IL-18: interleukin 18
AIFM1: apoptosis inducing factor mitochondria associated 1
GZMB: granzyme B
CHMP3: charged multivesicular body protein 3
TME: tumor microenvironment
NGS: next-generation sequencing
ACD: accidental cell death
RCD: regulated cell death
PCD: programmed cell death
KM: Kaplan-Meier
ROC: receiver operating characteristic
AUC: area under curve
KEGG: Kyoto Encyclopedia of Genes and Genomes
GO: Gene Ontology
TCGA: The Cancer Genome Atlas
TIIC: tumor-infiltrating immune cell
ssGSEA: single sample Gene Set Enrichment Analysis
TCIA: the Cancer Immunome Atlas
TIDE: Tumor Immune Dysfunction and Exclusion
HLA: human leukocyte antigen
IC50: half-maximal inhibitory concentration
Scissor: Single-Cell Map of Diverse Immune Phenotypes in the Breast Tumor Microenvironment
GDSC: Genomics of Drug Sensitivity in Cancer
GPCR: G protein-coupled receptor
TCR: T cell antigen receptor
TOM: Topological Overlap Matrix
