# Supplementary material for: Identification of a PANoptosis-related Gene Signature for Predicting the Prognosis, Tumor Microenvironment and Therapy Response in Breast Cancer
Source: J Cancer. 2024 Jan 1;15(2):428–43. doi: 10.7150/jca.90113 (PMC10758028; doi:10.7150/jca.90113)
Supplement: Supplementary file 1 — Supplementary figures and tables. [file jcav15p0428s1.zip › Supplementary materials/Supplementary Figures and Table S1-2.pdf]

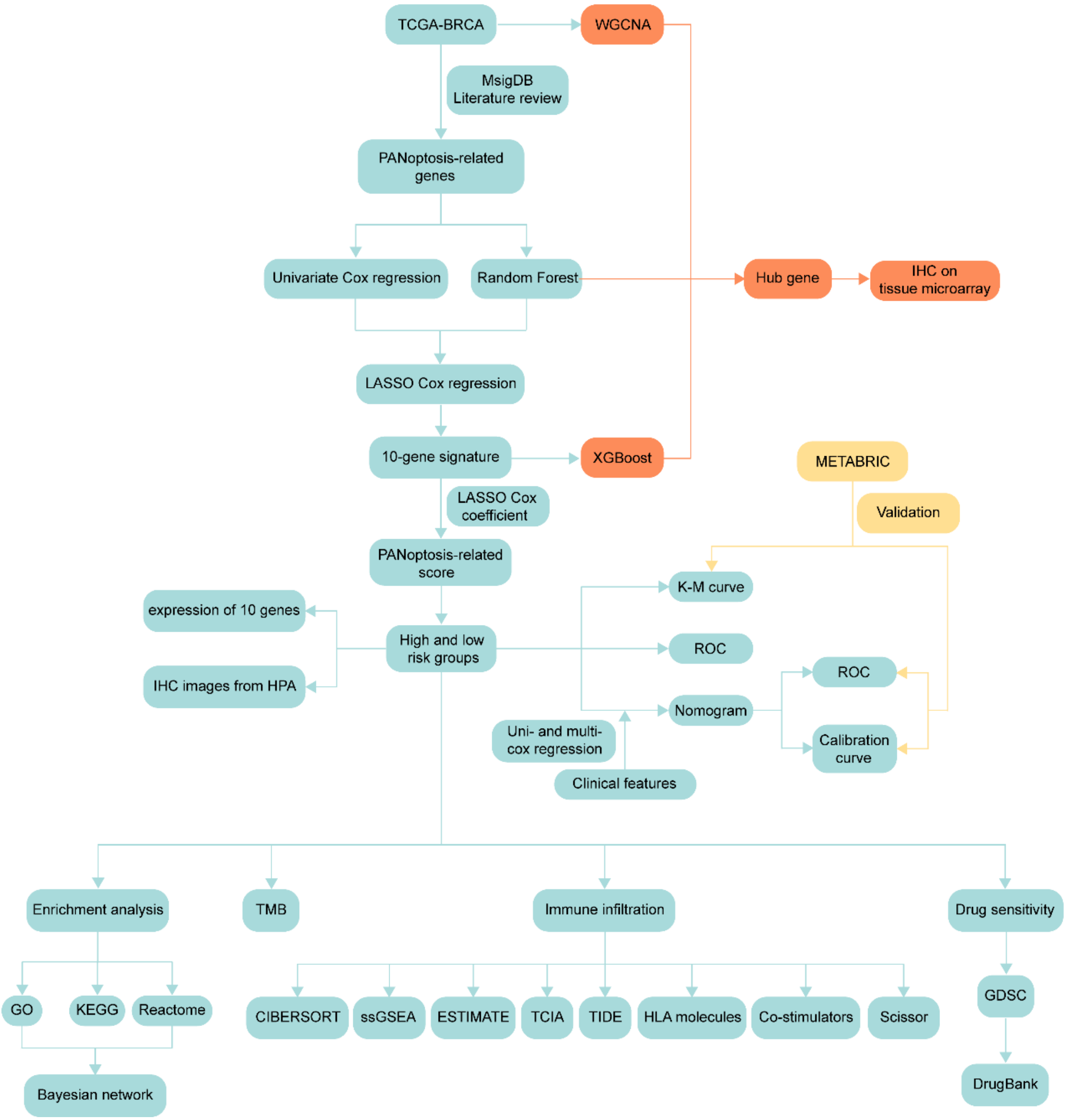

Figure S1. Workflow of this study.

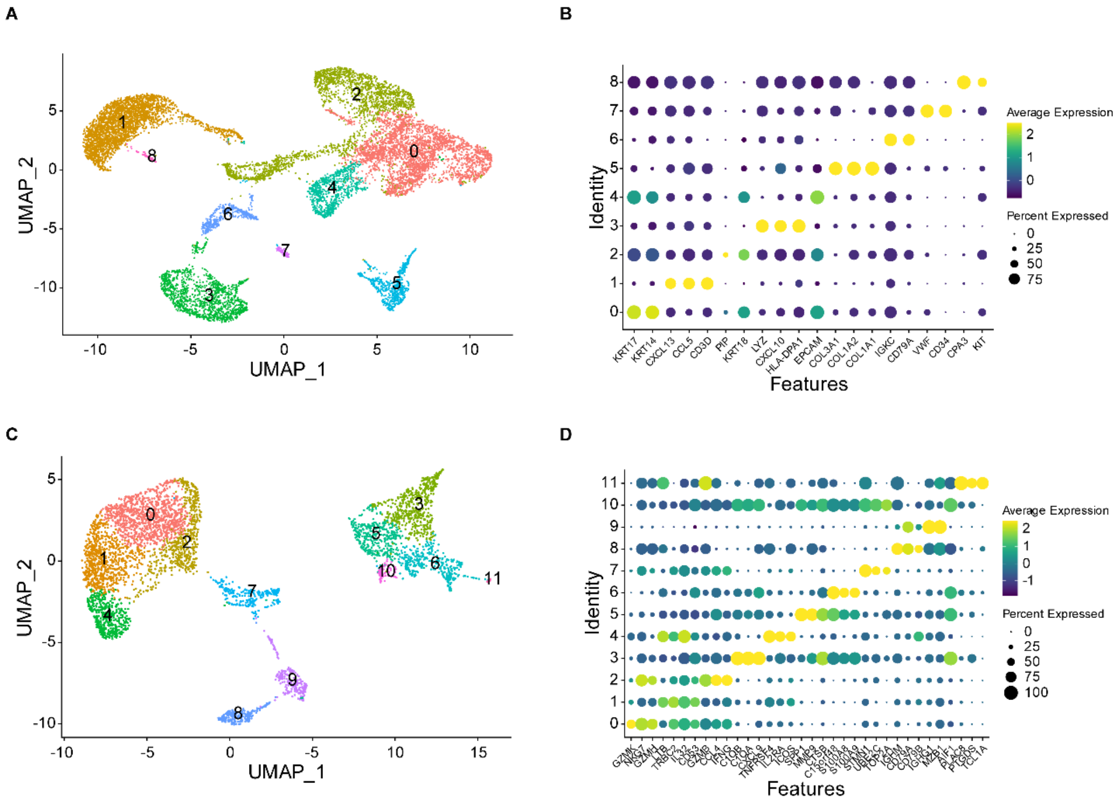

Figure S2. Demonstration of single cell clusters and marker genes. (**A**) UMAP visualization of cell clusters. (**B**) Visualization of marker gene expression. (**C**) UMAP visualization of cell clusters among extracted immune cells. (**D**) Visualization of marker gene expression among extracted immune cells.

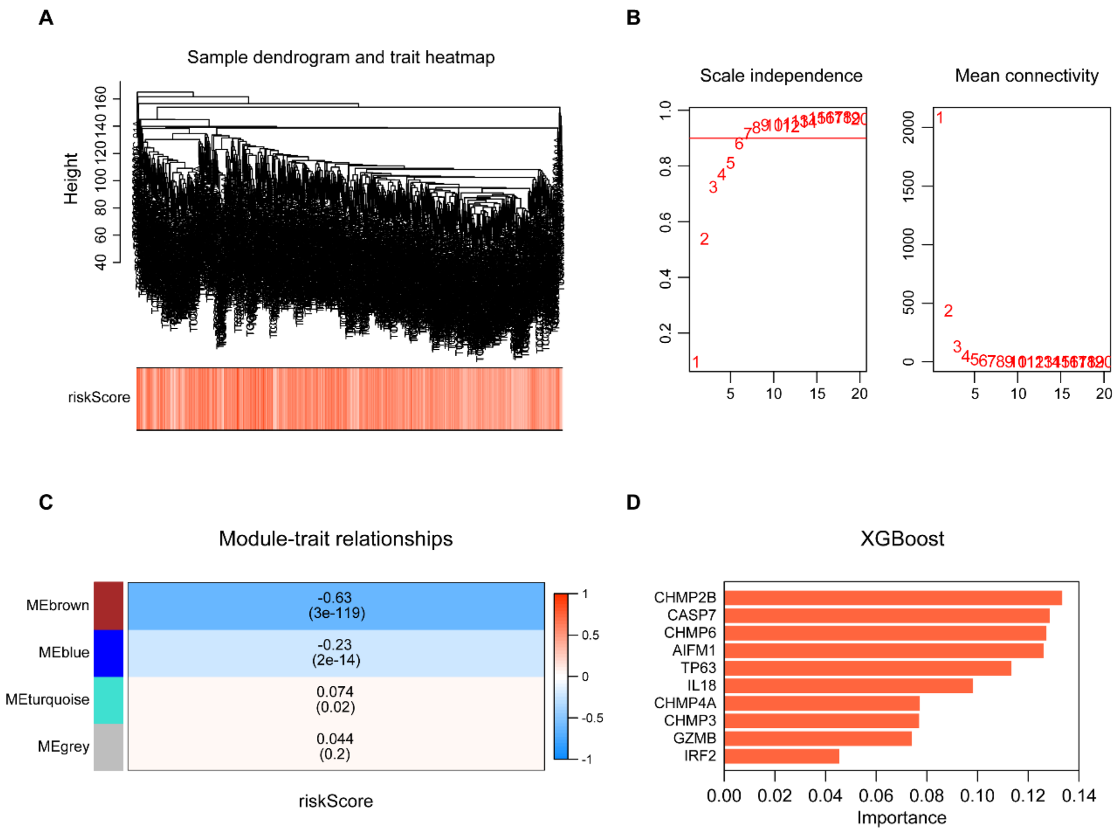

Figure S3. Construction of the weighted co-expression network. **(A)** Clustering dendrogram of patients in the TCGA-BRCA dataset and heatmap of risk score. Regarding the color in the heatmap, red means a higher risk score and white means a lower risk score. **(B)** Analysis of the scale-free fit index (the left panel) and the mean connectivity (the right panel) for various soft-thresholding power values. **(C)** Correlation between the modules and risk score. Red represents the positive correlation and blue represents the negative correlation. **(D)** Importance of each model gene in the XGBoost algorithm.

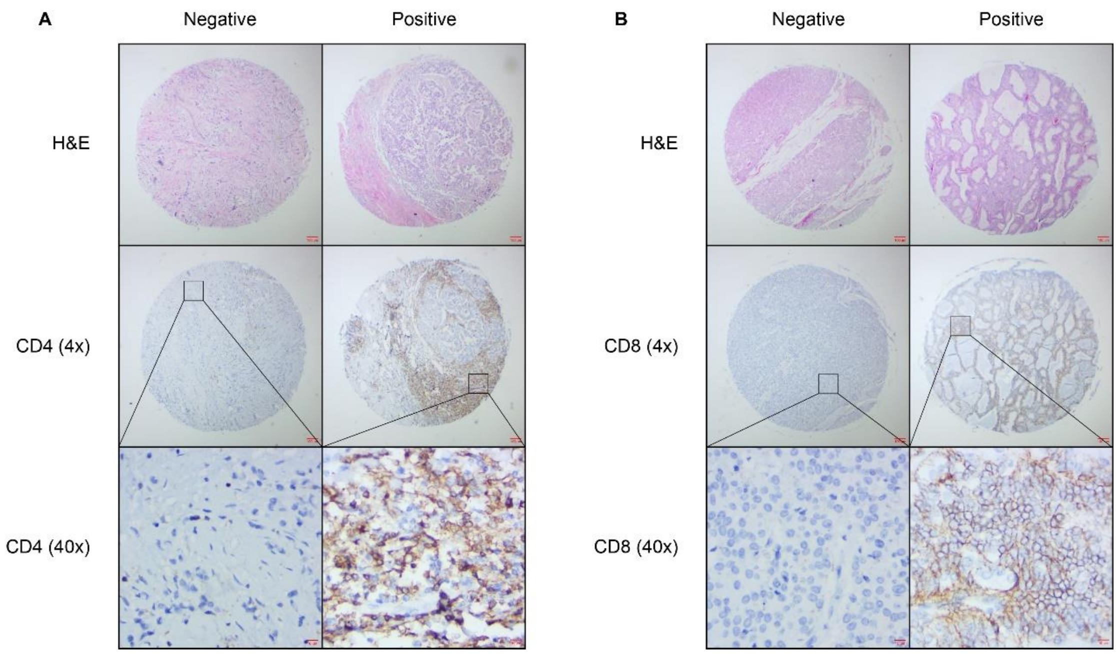

Figure S4. Representative images of IHC analysis. Representative images of CD4 expression (**A**) and CD8 expression (**B**) in tumor tissue. IHC, immunohistochemistry.

Table S1. Baseline characteristics of BC patients in TCGA, METABRIC and our cohorts.

**Variables**	**TCGA (n = 855)**		**METABRIC (n = 1848)**		**Our Cohort (n = 105)**		**p-value**
	[a]N	%	N	%	N	%	
Age, years							< 0.001
< 50	245*	28.7	398*	21.5	58*	55.2	
≥ 50	610*	71.3	1450*	78.5	47*	44.8	
ER status							
Negative	302	35.3	423*	22.9	36	34.3	< 0.001
Positive	553	64.7	1425*	77.1	69	65.7	
T stage							0.001
T1	229	26.8			46	43.8	
T2	510	59.7			54	51.4	
T3	90	10.5			4	3.8	
T4	26	3.0			1	1.0	
[b]N status							0.108
Negative	428	50.1	946	51.2	64	61.0	
Positive	427	49.9	902	48.8	41	39.0	

BC, breast cancer; TCGA, The Cancer Genome Atlas; [a]N, number; %, percentage; ER, estrogen receptor; *, has statistical difference compared with other groups; [b]N, lymph node.

Table S2. Sixty-five PANoptosis-related genes.

Gene	Category
TNF	Necroptosis
TLR3	Necroptosis
RIPK3	Necroptosis
RIPK1	Necroptosis
MLKL	Necroptosis
FASLG	Necroptosis
FAS	Necroptosis
FADD	Necroptosis
BAK1	Pyroptosis
BAX	Pyroptosis
CASP1	Pyroptosis
CASP3	Pyroptosis
CASP4	Pyroptosis
CASP5	Pyroptosis
CHMP2A	Pyroptosis
CHMP2B	Pyroptosis
CHMP3	Pyroptosis
CHMP4A	Pyroptosis
CHMP4B	Pyroptosis
CHMP4C	Pyroptosis
CHMP6	Pyroptosis
CHMP7	Pyroptosis
CYCS	Pyroptosis
ELANE	Pyroptosis
GSDMD	Pyroptosis
GSDME	Pyroptosis
GZMB	Pyroptosis
HMGB1	Pyroptosis
IL18	Pyroptosis
IL1B	Pyroptosis
IL1A	Pyroptosis
IRF1	Pyroptosis
IRF2	Pyroptosis
TP63	Pyroptosis
AIFM1	Apoptosis
AKT3	Apoptosis
APAF1	Apoptosis
APPL1	Apoptosis
BMF	Apoptosis
BNIP3L	Apoptosis
BOK	Apoptosis
CASP10	Apoptosis
CASP2	Apoptosis
CASP6	Apoptosis
CASP7	Apoptosis
CASP8	Apoptosis
CD14	Apoptosis
CHUK	Apoptosis
CRADD	Apoptosis
DFFA	Apoptosis
DFFB	Apoptosis
E2F1	Apoptosis
HMGB2	Apoptosis
IGF1	Apoptosis
LY96	Apoptosis
PPP3R1	Apoptosis

TFDP1	Apoptosis
TICAM1	Apoptosis
TNFRSF1A	Apoptosis
TNFSF10	Apoptosis
TP73	Apoptosis
TRAF2	Apoptosis
UACA	Apoptosis
UNC5B	Apoptosis
YWHAE	Apoptosis
YWHAG	Apoptosis
